# Protective efficacy of menthol propylene glycol carbonate compared to N, N-diethyl-methylbenzamide against mosquito bites in Northern Tanzania

**DOI:** 10.1186/1756-3305-5-189

**Published:** 2012-09-05

**Authors:** Eliningaya J Kweka, Stephen Munga, Aneth M Mahande, Shandala Msangi, Humphrey D Mazigo, Araceli Q Adrias, Jonathan R Matias

**Affiliations:** 1 Division of Livestock and Human Diseases Vector Control, Mosquito section, Tropical Pesticides Research Institute, P.O.BOX 3024, Arusha, Tanzania; 2 Department of Medical Parasitology and Entomology, Catholic University of Health and Allied Sciences, P.O. Box 1464, Mwanza, Tanzania; 3 Centre for Global Health Research, Kenya Medical Research Institute, P.O. Box 1578–40100, Kisumu, Kenya; 4 Poseidon Science Foundation, 122 East 42nd Street, Suite 1700, New York, 10168, NY, USA

## Abstract

**Background:**

The reduction of malaria parasite transmission by preventing human-vector contact is critical in lowering disease transmission and its outcomes. This underscores the need for effective and long lasting arthropod/insect repellents. Despite the reduction in malaria transmission and outcomes in Tanzania, personal protection against mosquito bites is still not well investigated. This study sought to determine the efficacy of menthol propylene glycol carbonate (MR08), *Ocimum suave* as compared to the gold standard repellent N, N-diethyl-methylbenzamide (DEET), either as a single dose or in combination (blend), both in the laboratory and in the field against *Anopheles gambiae s.l* and *Culex quinquefasciatus.*

**Methods:**

In the laboratory evaluations, repellents were applied on one arm while the other arm of the same individual was treated with a base cream. Each arm was separately exposed in cages with unfed female mosquitoes. Repellents were evaluated either as a single dose or as a blend. Efficacy of each repellent was determined by the number of mosquitoes that landed and fed on treated arms as compared to the control or among them. In the field, evaluations were performed by human landing catches at hourly intervals from 18:00 hr to 01:00 hr.

**Results:**

A total of 2,442 mosquitoes were collected during field evaluations, of which 2,376 (97.30%) were *An. gambiae* s.l while 66 (2.70%) were *Cx. quinquefaciatus*. MR08 and DEET had comparatively similar protective efficacy ranging from 92% to 100 for both single compound and blends. These findings indicate that MR08 has a similar protective efficacy as DEET for personal protection outside bed nets when used singly and in blends. Because of the personal protection provided by MR08, DEET and blends as topical applicants in laboratory and field situations, these findings suggest that, these repellents could be used efficiently in the community to complement existing tools. Overall, *Cx. quinquefasciatus* were significantly prevented from blood feeding compared to *An. gambiae* s.l.

**Conclusion:**

The incorporation of these topical repellents for protection against insect bites can be of additional value in the absence or presence of IRS and ITNs coverage. However, a combination of both the physical (bed nets) and the repellent should be used in an integrated manner for maximum protection, especially before going to bed. Additional research is needed to develop repellents with longer duration of protection.

## Background

Mosquitoes are one of the major disease vectors affecting human populations worldwide. The main approaches to reducing human-vector contact include: use of physical barriers, such as bed nets [1,2]; chemical barriers, such as indoor residual spray [2]; topical application of repellents and burning insect repellent plants indoors [3-6]; house modification; [7] and other behavioral mechanisms such as zoo-prophylaxis [8]. Despite reports of reduction in the burden of malaria disease and its vectors in some African states [9,10], other personal protection methods against mosquito bites are still needed to complement the existing tools that have contributed to this declining trend. Insect repellents are one of the major sources for personal human protection against mosquito bites. An insect repellent has been defined as a substance applied to the skin [4], clothing [11] or other surfaces which discourage or inhibit insects (arthropods in general) from landing or climbing on that surface and sometimes with short or long range spatial repellence. The widely available standard approved synthetic topical repellent is DEET (N, N-diethyl-methylbenzamide), while a large number of plant based repellents are evolving. DEET has been referred to as the gold standard repellent since it has been widely marketed as a commercial repellent and showed protection efficiency of 8 hours after application [12]. Since its first use in 1954, DEET has been shown to act as a strong molecular confusant by jumbling the insect’s odour receptors activity in different concentrations both in the field and laboratory [13,14]. Plant based arthropod repellents currently in the market includes Citronella (*Cymbopogonnardus* or *Cymbopogonwinterianus*) [15] and lemon eucalyptus (*Eucalyptus maculate citiodon*) [16]. Since ancient times, plant products or whole plants have been used to repel or kill mosquitoes and other domestic insect pests in communities [3-6]. The methods of delivering these traditional repellents were thermal expulsion and direct burning, which have been demonstrated to cause reduction in indoor density of mosquitoes [5,6]. In other instances the essential oils from plants have been shown to last longer and have higher protective efficacy when mixed with a carrier, such as a cream, than synthetic repellents mixed with a carrier such as gylcerin [17,18]. In Tanzania, *OcimumsppLantana camara*,neem ( *Azadirachtaindica*), *Eucalyptus spp* and other plant species have efficiently repelled and killed the main malaria ( *An.gambiae* s.s, *An.arabiensis)* and non-malaria vectors( *Cx. quinquefasciatus)*[3,4,16]. Essential oils from these plant materials such as *Ocimum kilimandscharicum*[4], *O. suave*[4], *L. camara*[4,19], *A. indica*[20] and *O. forskolei*[21] have different chemicals in varying compositions. The variations in the protective efficacy of essential oils in different mosquito species has been attributed to differences in the concentrations of chemical constituents [3,4,21]. Menthol propyleneglycol carbonate(MR08), a derivative of naturally occurring menthol, has shown higher feeding inhibition in laboratory tests against *Aedes aegypti* and other arthropods, such as sand flies [22]. However, MR08 has not been evaluated against *Cx. quinquefasciatus* and *An.gambiae* s.l *..* In sand flies, time to first landing was greater than 120 minutes post application with MR08 [22].

The current study assessed the repellent bioactivity of MR08 plant based product, *O.suave* as compared to DEET against *An.gambiae* s.s, *An.arabiensis* and *Cx. quinquefasciatus* in both laboratory and field conditions.

## Methods

### Recruitment of volunteers

Volunteers were given informed consent forms to read in order to clearly understand the study objectives and this was further re-enforced by discussions with the study teams. Those who participated in the field trials were screened for malaria parasites before being recruited into the study. Malaria parasite screening was done on a weekly basis for all participants during the course of study.

### Inclusion criteria

All study participants were above 18 years of age, agreed to sign the consent form and were screened for malaria parasites before participating in the study. All mosquitoes used for evaluations in the laboratory were 3 days old post emergence and were non-blood fed.

### Exclusion criteria

All persons below 18 years of age and those who did not sign the consent form and not screened for malaria parasites were excluded.

### Study design

The study had two designs: controlled laboratory-based experiments and also small scale community based field trials. Laboratory study designs had five replicates in each dosage. The field study was conducted using a 5 by 5 Latin square design. During the study period, treatments were rotated in each house among the selected houses to avoid positional bias. Additionally, wind speed was recorded daily and categorized as normal, moderate or strong.

Laboratory study was conducted at The Tropical Pesticides Research Institute (TPRI)(Arusha, Tanzania), while the field trials were carried out in Mabogini village around Lower Moshi irrigation schemes, 10 kilometers south of Moshi town, Tanzania. This area is known to have high mosquito densities mostly throughout the year compared to other areas [23].

### Concentration and blend preparations

Three repellents, MR-08, DEET and *O. suave,* were formulated at different dosages while DEET was used as the gold standard repellent [12] and compared to two botanical based repellents, MR08 and *O. suave* plant extracts. MR08 is menthol propyleneglycol carbonate [24] while *O.suave* extracts were made by steam distillation from *O.suave* shrubs. The active ingredients of the *O.suave* extracts have been described elsewhere [25]. DEET is well known as an effective repellent in different studies [12,26]. The dosages were made singly at a dosage of 10, 20 and 30% by volume with base cream as carrier substance for all the three repellent formulations. The base cream is a commercially available cold cream base (Rite Aid Corp, Harrisburg, PA, USA) comprised of mineral oil, water, beeswax, ceresin, sodium borate, fragrance and carbomer. Combination of two repellents at the lowest dosages where made (hereinafter referred to as blends). Three blends were made (MR08 10% + DEET 10%, hereinafter named - blend 1); (MR08 10% + *O.suave* 10%, hereinafter referred to as blend 2) and ( *O.suave* 10% + DEET 10%, hereinafter named blend 3). Both single dosage and blend were then evaluated in the laboratory and in the field. These dosages were made in volume ratio of repellent and base cream as a carrier (Repellent: Cream base ratios at 30:70; 20:80 and 10:90).

### Cage repellent evaluation

**Figure 1:**
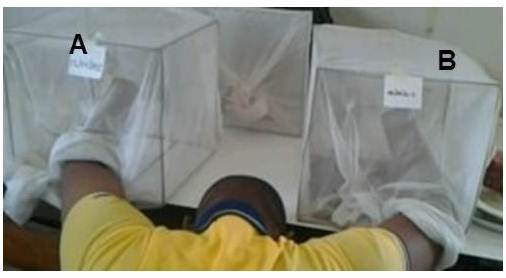
Picture showing a volunteer evaluating feeding succession in a (A) treatment and (B) Control.

Two mosquito species were used in the laboratory experiments: *Cx.quinquefasciatus* (Mabogini strain) and *An.gambiae* s.s (Kisumu strain). Twenty five mosquitoes which were 3 days old post emergence [27] were used for these experiments; sugar solution (Sucrose 10%) was taken out from the cages 30 minutes before trials. The two arms of the same individual were used, with one arm acting as treatment (applied with 2 ml of repellent on the skin surface) while the other arm was used as control and applied with base cream on the surface. The two arms of the same individual were used simultaneously to avoid bias occasioned by differential attractiveness (Figure 1) [28]. A timer was set after introducing mosquitoes into a cage and stopped after a mosquito landed on a treated or untreated arm. The blood fed mosquitoes were scored after 30 minutes based on the abdominal status as described in The WHO protocol [27]. These experiments were conducted in standard cages of 30 cm x 30 cm x 30 cm.

### Field evaluation of repellents

Two repellents, MR08 30% and DEET 30% were selected as single dosages for field evaluations, while blends used were blend 2 and blend 3. Each single repellent or blend was evaluated using a pair of volunteers who exposed their legs treated with either base cream alone (control) or repellent in base cream [27]. Five houses were used in 5 × 5 Latin square designs with a total of ten volunteers participating in these field trials. Evaluations were conducted outdoors in Mabogini village during the season when farmers were transplanting rice paddies and hence higher mosquito densities. Mosquitoes were collected using a mechanical hand aspirator with assistance of a hand held battery torch. Mosquitoes were collected at hourly intervals from 18:00 hr to 01:00 hr. The mosquitoes which were caught using the aspirators were taken to the field station laboratory and sorted using morphological keys to species level and separated by sexes as described by Gillies and Coetzee [29] under a dissecting microscope.

Houses selected for mosquito sampling were 500 meters apart and all houses were made of burnt bricks with an iron sheet roof. Wind speed and other parameters, such as presence or absence of rain were recorded.

### Statistical analysis

Data analysis was performed using the PWAS statistics 18.0 (SPSS Inc., Chicago, IL). The comparison between control and treatments was carried out using a paired t-Test with two samples of equal variance (homoscedastic). The comparison of the mean time taken to the first landing in treated and control host was done using one-way analysis of variance (ANOVA).

Analysis of field data was done using multi-factorial analysis of variance (MANOVA). The Tukeys HSD test was applied to assess the contribution of each factor to the mosquito species density sampled by the volunteers, such as days, wind speed and house locations. Students paired t-test homoscedastic was used to compare the overall protection difference between blend treatments evaluated in the field.

### Ethical issues

This study was given an ethical approval from KCM College of Tumaini University and Tropical Pesticides Research Institute Research Ethics Review Committee. All volunteers were given written consent forms signed in front of a witness who was not a study participant. All volunteers were screened for malaria parasites before the study and weekly for the period of the study. None of the volunteers were found to be infected with malaria parasites in the field study.

## Results

### Laboratory evaluations of repellents

**Figure 2:**
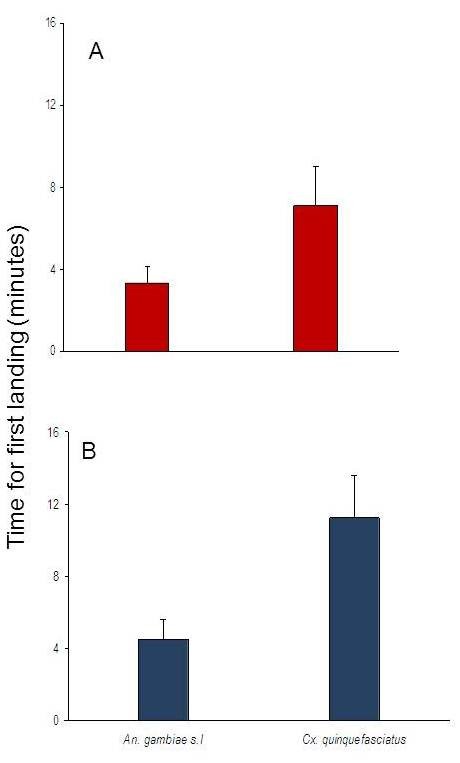
Time taken in (A) laboratory and (B) field first biting for *An.gambiae* s.l and *Cx. quinquefasciatus.*

Seven dosages of repellents were evaluated singly and in three blends. Among the repellents evaluated singly, *O. suave* had the shortest time of 2.20 minutes before mosquitoes landed for blood feeding, while using the blends, the least time to landing on the volunteer’s arm was 1.90 minutes given by Blend 1. Overall, more protection time was observed with *Cx. quinquefasciatus* compared to *An. gambiae* for first landing (Figure 2).

**Figure 3:**
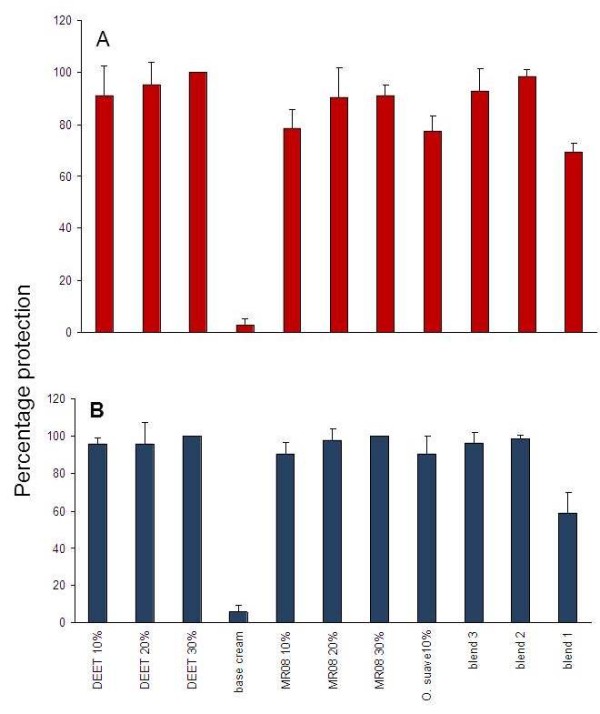
Protection efficiency of single dosage and blend evaluated in the laboratory for (A) *An.gambiae* s.l and (B) *Cx. quinquefasciatus.*

The mean protection efficacy of the singly evaluated dosages ranged from 77.60% to 100% while for blends ranged from 58.80% to 98.80% in the laboratory (Figure 3). The protection from each treatment was significantly different for both *An. gambiae* s.s and *Cx. quinquefasciatus* relative to the control (Table 1).

**Table 1:** Proportions of *An. gambiae* s.s and *Cx. quinquefasciatus* protected from feeding on arms treated with different repellent concentrations and controls in laboratory

**Species**	**Repellent**	**% Concentrations**	**Treatments**	**Control**	**Paired T-test**
*An. gambiae* s.s	DEET	10	91.2	2.7	t = 19.64,*P <* 0.001
		20	95.2	3.6	t = 25.57, *P <* 0.001
		30	100	4.8	t =112.99, *P <* 0.001
	MR08	10	78.4	5.6	t = 35.02, *P <* 0.001
		20	90.4	3.7	t = 20.28, *P <* 0.001
		30	91.2	2.9	t =40.95, *P <* 0.001
	OS	10	77.4	3.8	t = 45.19, *P <* 0.001
	Blend 1	10	69.4	2.9	t = 43.35, *P <* 0.001
	Blend 2	10	92.8	4.8	t = 70.09, *P <* 0.001
	Blend 3	10	98.4	3.4	t =24.32, *P <* 0.001
*Cx. quinquefasciatus*	DEET	10	95.6	2.7	t = 61.39, *P <* 0.001
		20	96.1	4.4	t = 18,59, *P <* 0.001
		30	100	6.9	t =75.91, *P <* 0.001
	MR08	10	90.4	5.8	t = 33.29, *P <* 0.001
		20	97.8	4.9	t = 39.23, *P <* 0.001
		30	100	6.7	t =75.91, *P <* 0.001
	OS	10	90.2	5.9	t = 32.22, *P <* 0.001
	Blend 1	10	58.8	5.3	t = 13.42, *P <* 0.001
	Blend 2	10	98.8	4.9	t = 71.33, *P <* 0.001
	Blend 3	10	96.0	6.1	t = 31.23, *P <* 0.001

The protective efficacy of MR08 10% was statistically insignificant (P = 0.347) when compared to DEET 10%; MR08 20% and DEET 20% using *An.gambiae* s.s. Similarly, MR08 30% gave significantly less protection than DEET 30% in volunteers (Table 2). However, comparative evaluation between the different blends against *An. gambiae* s.s showed that Blend 2 gave significantly higher protective efficacy than blend 1 (t = 2.78, df = 4, *P* ≤ 0.001); Blend 3 gave significantly higher protection than blend 1 ( *P* = 0.002) while protective efficacy between Blend 2 and blend 3 was statistically insignificant ( *P* =0.180).

**Table 2:** Comparative protection of DEET and MR08 repellents against *An.gambiae* s.l and *Cx. quinquefasciatusat* different dosages

**Species**	**% Concentration**	**DEET**	**MR08**	**Paired T-test**
*An. gambiae* s.s	10	91.2	78.4	t = 2.43, *P =* 0.036
	20	95.2	90.4	t = −271.33, *P =* 0.054
	30	100	91.2	t = 5.88, *P =* 0.002
*Cx. quinquefasciatus*	10	95.6	90.4	t = 1.87, *P =* 0.067
	20	96.1	97.8	t = 0.42, *P =* 0.347
	30	100	100	NS*

Note: NS* mean not significant different and results could not be displayed in PWAS statistics output.

*Cx. quinquefasciatus* feeding protection was performed using dosages made singly;MR08 10%, DEET 10%, MR08, 20%,DEET 20%,MR08 and DEET 30% which in comparisons had no significant differences in protection efficacy (Table 2).

The results of comparative protection between *An. gambiae* s.s and *Cx. quinquefasciatus* in different dosages, indicated that in MR08 10% more *Cx. quinquefasciatus* were prevented from feeding than *An. gambiae* s.s but this was statistically insignificant (P > 0.05). Experiments conducted with DEET 10%, indicated that both *Cx. quinquefasciatus* and *An. gambiae* s.s were equally inhibited from feeding but were statistically insignificant too. ForMR08 20%, both *Cx. quinquefasciatus* and *An. gambiae* s.s were equally inhibited from feeding and were not statistically significant. For DEET 20% both *Cx. quinquefasciatus* and *Angambiae* s.s showed equal proportions of feeding inhibition and were not significantly different. Using MR08. 30%, *Cx. quinquefasciatus* were significantly inhibited from feeding compared to *An. gambiae s.s*. *Cx. quinquefasciatus* and *An. gambiae* s.s were not statistically different in feeding protection against DEET 30%.They were 100% protected from feeding. OS protected significantly more *Cx. quinquefasciatus* from feeding than *An. gambiae* s.s and this was significantly different (Table 3). The protective efficacy between blend 2 and 3 was statistically insignificant, however Blend 1 showed significantly higher protection, with *An. gambiae* s.s being inhibited more from feeding compared to *Cx. quinquefasciatus* (Table 3).

**Table 3:** Percentage of *An.gambiae* s.s and *Cx. quinquefasciatus* repelled from feeding on treated arms in laboratory trials

**Repellent**	**% Concentration**	***An.gambiae*s.s**	***Cx. quinquefasciatus***	**Paired T-test**
DEET	10	91.2	95.6	t = 1.06, *P =* 0.175
	20	95.2	96.1	t = 0.209, *P =* 0.422
	30	100	100	NS*
MR08	10	78.4	90.4	t = 2.78, *P =* 0.025
	20	90.4	97.8	t = 1.69, *P =* 0.083
	30	91.2	100	t = 5.88, *P =* 0.002
OS	10	77.4	90.2	t = 4.38, *P =* 0.006
Blend 1	10	69.4	58.8	t = 2.82, *P =* 0.036
Blend 2	10	92.8	98.8	t = 0.39, *P =* 0.359
Blend 3	10	98.4	96.0	t = 1.55, *P =* 0.098

Note: NS* mean not significant different and results could not be displayed in PWAS statistics output.

### Field evaluations

After laboratory evaluations, use of blend 1 was discontinued due to the poor performance observed in protection against both *An. gambiae* s.s and *Cx. quinquefasciatus*. Blend 2 and 3 were used in field trials together with MR08 30% and DEET 30% against wild mosquito populations.

A total of 2,442 mosquitoes were collected during field evaluations. A total of 600 (24.57%), mosquitoes were collected by volunteers from treatment groups, while 1,842 (75.43%), were from control groups. Out of the mosquitoes collected, 97.30% (2376/2442), were *An. gambiae* s.l and 2.70% (66/2442) were *Cx. quinquefaciatus.*

**Figure 4:**
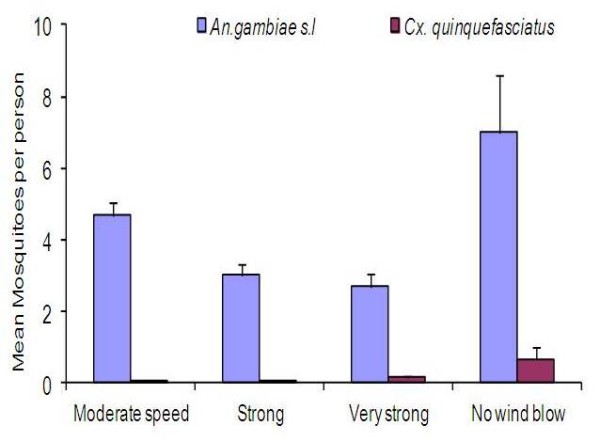
The effect of wind speed on the number of caught (A) *An.gambiae* s.l and (B) *Cx. quinquefasciatus* densities during field evaluation of repellents.

**Figure 5:**
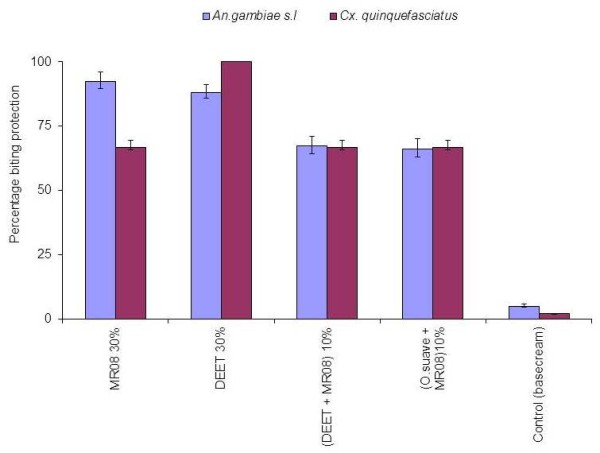
The mean protection of single repellents and blends for (A) *An.gambiae* s.l and (B) *Cx.quinquefasciatus*in field situation.

**Figure 6:**
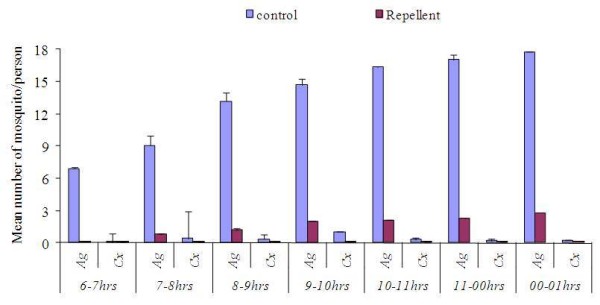
Effect of repellents on mean mosquito densities caught in treatments and controls for 7 hours.

In the analysis, the four repellents evaluated had significant vector reduction in comparison to controls for both *An. gambiae* s.l (F = 195.95, df = 4, *P <* 0.001) and *Cx. quinquefasciatus* (F = 8.861, df = 4, *P <* 0.001). Overall, when mosquitoes were taken as independent variables, house position had no effect on repellent efficacy since houses had no mosquito density variation (F = 0.786, df. = 4, *P =* 0.672). The density of mosquitoes sampled from all houses was statistically insignificant when wind speed categories was used as a grouping factor (F = 0.624, df. = 4, *P* = 0.645). Despite variations in wind speed during the sampling days, there were no statistical differences in mosquito densities (Figure 4). Tukeys HSD test analysis for all the dosages used showed that the protective efficacy was dosage dependent (Figure 5). Differences in protective efficacies against *An. gambiae* s.l and *Cx. quinquefasciatus* were also noted among the single repellent and the blends *.* The treatments were able to reduce the mosquito density on treated volunteers relative to control in the evening biting cycles (Figure 6). *Cx. quinquefasciatus* took significantly longer time to land and probe on the volunteers than *An. gambiae* s.l both under laboratory and field conditions (F = 15.42, df = 1, *P <* 0.001) (Figure 2).

## Discussion

This study has demonstrated that MR08had nearly the same results compared to the gold standard repellent DEET in both laboratory and field situations. In the laboratory, DEET 30% (v/v) had protection efficiencies of 100% for the two mosquito species while MR08 had a protection efficiency of 100% and 91.2% for *Cx. quinquefasciatus* and *An. gambiae* s.s respectively, against mosquito bites 1 hour post exposure. Under field conditions, 30% MR08 had the highest protection efficiency (92.39%) while DEET 30% had a protection efficiency of 88.17% for seven hours (from 18:00 hrs to 01:00 hrs). These hourly intervals coincided with the first effective host seeking cycles of malaria vectors, *An. gambiae* s.l. In both laboratory and field evaluation of various repellent dosages, protection was found to be dosage dependent as found elsewhere [30,31] while with blends, the synergistic effect was dosage mixture dependent [11,32]. The strategy of using repellents in combination (Blends) conferred a better protection with little amount of repellent used (Blend 2 and Blend 3) compared to DEET and MR08 30% alone. Both blend 2 and 3 protected volunteers by 82 and 85%, and 98.4 and 92.8% in the field and laboratory respectively up to a period of 7 hours. Mosquito densities and efficacy of compound evaluated in the field had the same environmental variability, such as wind and house locations, thus these findings are likely to be as a result of the chemical compounds in the repellents. The application of repellents reduced the density of mosquito landing on the treatment group compared to the control volunteers (Figure 4).

In northern Tanzania, high distribution and usage of ITNs have shown increased protection to communities against malaria vectors when more than 80% of the population own and use bed nets properly [33,34]. In other studies, bed net utilization has proved to reduce malaria infections when used properly as personal protection tools [35,36]. There is increased exposure risk to those who are out of bed net either in the evening or morning during the peak biting cycles of the malaria vectors [37]. Due to high coverage of ITN and IRS programmes, malaria vector feeding and resting behaviours’ are likely to have changed to maximize available feeding opportunities. Reports suggest that *An. gambiae* s.s has changed from being endophagic and endophilic to exophagic and exophilic respectively [2,24,38,39]. This behavioural adaptation may present a problem in the personal protection in individuals when outdoors [39].

Application of MR08 as topical repellent could reduce the biting risk to those outdoors, hence adding protective value for individuals who are outside the bed nets during earlier and late biting cycles. These findings suggest that the protective efficacy which is maintained for a period of 7 hours is believed to be realistic for users who retire late to bed under the protection of the bed net. Field evaluations of repellents (18:00 to 01:00 hrs) were conducted during the first host seeking cycles of mosquitoes [40]. Therefore, one can extrapolate the efficacy of these products in protecting individuals against random opportunistic host seeking mosquitoes. Thus, MR08 can complement the effects of ITNS and IRS for the unprotected individuals when used as the topical repellent [41]. Currently in western Kenya, the decrease of relative abundance of vector species have been observed due to high implementation of intervention tools against malaria vectors [42] while in other places the displacement of the vector species composition have been reported [43]. In Dar-es-salaam Tanzania, outdoor feeding among malaria vectors has been reported to be increasing due to wide ITNs coverage, hence increasing biting pressure on unprotected individuals outdoors [44].

In the current study, the observed reduction in biting rates both in laboratory and field evaluations may have great impact on infective bite reduction when incorporated with wide use of IRS and ITNs in the community. Malaria decline in different parts of Africa is associated with high ITN and IRS coverage [45,46] and reliable diagnostic and treatment services [45]. On the other hand, it has been observed that, when vector density declines, communities have a tendency to useless personal protection tools, such as bed nets, against disease vectors [47]. It is necessary for the community to become more aware of using topical repellents.

In controlling African malaria vectors, ITN and IRS have been deployed with the assumption that vector behaviour remains endophilic and endophagic. This assumption is derived from classical behavioural studies by Gillies and Coetzee [29]. These vectors have changed behaviour and tend to feed outdoors (exophagic) due to massive IRS, house modifications and ITN coverage [7,39,48]. In most malaria endemic areas, covering all households with IRS and ITNs is practically impossible [40,49]. Another important limitation of ITN and IRS is that many people do not retire indoors or to bed earlier and miss the benefit of the protection offered by these methods during the earlier biting cycles of malaria vectors [44]. Additional tools, such as repellents, should be considered to supplement existing vector protection tools.

Protection against infective bites from arthropods can be achieved by either avoiding infested areas, protective gear usage (cloth with repellents and ITNs), house modifications or applying topical insect repellents and use of IRS [7,11,50]. Most commercially available repellents and formulations have up to 40% DEET, which is preferred for use in areas with high biting pressure or environmental conditions that promote the loss of repellent on skin surface [51-53]. This amount of DEET (40%) seemed to be higher than MR08 blends (of 10% and 20%), which could reduce the production costs and be affordable to the large populations.

Thousands of plant resources have been tested as sources of insect repellents [4,11,20,54,55]. ITN, treated curtains and IRS coverage have critically reduced entomological inoculation rates [36,47] and integrating the evaluated MR08 repellents in reducing human-vector contact might further reduce EIR to even lower rates than currently reported at0.54 ib/trap/year in the study area [56]. But this reduction of EIR can only be estimated for the indoor biting mosquitoes and not for the outdoor biting ones, where other tools, such as ITN and IRS cannot be implemented [57]. Therefore, during outdoor movements and late retirement to bed, application of topical repellents should be emphasized [58], together with house design modification for indoor vector reduction [7]. MR08 and other repellents could be taken into consideration to fill the gap to reduce transmission rate during this unprotected time.

Currently, slow release and vaporization methods are being tested to enhance the effectiveness of MR08 in preventing mosquito bites inside the household.

The appropriate method of delivering MR08 repellent to be protective and effective for all house occupants for longer duration of time is still on-going.

## Conclusion

This study suggests that MR08 is an effective compound against bites from both malaria and filarial vectors for unprotected community members. The integrated vector control involving the conventional control tools and these repellents is necessary to enhance personal protection and significantly reduce human-vector contact.

## Competing interest

EJK, AMM, SM, EJK, AQA declare to have no competing interest. JRM holds a patent on use of MR08 as an insect repellent.

## Authors’ contribution

EJK and JRM conceived and designed the study, carried out data analysis and results interpretation. EJK and SM drafted the manuscript. AMM and SM did both laboratory and field data collection. FM, AMM, SM, EJK, AQA and JRM revised the manuscript. All authors approved the final version of the manuscript.
